# Social Security Access Challenges for Chinese Farmers Migrating to Urban Settings

**DOI:** 10.1093/geroni/igaa057.1860

**Published:** 2020-12-16

**Authors:** Hong Mi, Qiyini Ma

**Affiliations:** Zhejiang University, Hangzhou, Zhejiang, China

## Abstract

In 2018, China had 564 million rural people, accounting for 40% of the total population. However in 2018, the total number of rural workers immigrating to urban areas related to economic need reached 288 million. This represents an increase of 46 million since 2011. The Chinese government piloted a new rural insurance as an answer to pushing urbanization forward to support rural immigrants. Consequently since 2009, the number of urban and rural residents participating in the basic old-age insurance has been kept above 500 million, making it the largest basic old-age insurance system, covering the largest number of persons in the world. Due to this insurance, an estimated 515 million people were lifted out of poverty, of which 27.41 million were older adults. Challenges for the Chinese government include transforming the Chinese situation of absolute poverty to relative poverty, and improving the living standard of older adults.

